# Cystic Echinococcosis of Camels: 12S rRNA Gene Variation Revealed Changing Pattern of Genetic Diversity Within *Echinococcus granulosus* sensu lato in the Middle East and North/Sub-Saharan Africa

**DOI:** 10.3389/fvets.2020.00618

**Published:** 2020-09-17

**Authors:** Mansoureh Dehghani, Mohammad Ali Mohammadi, Sahel Hemmati, Saeid Nasibi, Sima Rostami, Majid Fasihi Harandi

**Affiliations:** ^1^Student Research Committee, Afzalipour School of Medicine, Kerman University of Medical Sciences, Kerman, Iran; ^2^Research Center for Hydatid Disease in Iran, Department of Parasitology, Kerman University of Medical Sciences, Kerman, Iran; ^3^Dietary Supplements and Probiotic Research Center, Alborz University of Medical Sciences, Karaj, Iran

**Keywords:** hydatid disease, *Echinococcus intermedius*, *Echinococcus canadensis*, 12S rDNA, genetic variation, *Camelus dromedarius*, strain, MENA

## Abstract

Cystic echinococcosis (CE) is one of the most widespread zoonotic diseases, with considerable public health and economic importance. Camels play a significant role in transmission cycle of *Echinococcus granulosus* especially, in the Middle East and North Africa (MENA). The present study aimed to identify the genetic variation and haplotype distribution of camel isolates of *E. granulosus* sensu lato using all existing *E. granulosus* mitochondrial DNA data from camels in different parts of the world. Sequence data from 1,144 camel isolates of *E. granulosus* s.l. available in the NCBI GenBank including 57 camel hydatid cysts collected in central Iran were used to analyze the nature of genetic variation within the camel isolates of *E. granulosus* s.l. in MENA region. Fifty-seven camel isolates were also PCR-sequenced on mitochondrial 12S rRNA gene. Haplotype network analysis revealed seven different haplotypes clustered into four major groups. *E. intermedius* G6 was identified as the most commonly represented genotype in camels followed by G1. Mitochondrial 12S rRNA gene sequence analysis on 57 camel isolates identified three different genotypes, including *E. intermedius*/G6 (35/57, 61.4%), *E. granulosus* sensu stricto/G1-G3 (21/57, 36.8%) as well as one isolate identified as *E. ortleppi*/G5 (1/57, 1.8%). The number of base substitutions per site over 420 positions of partial 12S rRNA gene sequences were shown as 0.000 and 0.004 for *E. intermedius* (G6) corresponding to the Middle East and sub-Saharan isolates, respectively. Camel isolates of *E. granulosus* in the MENA region present moderate genetic diversity (Hd = 0.5540–0.6050). The Middle East isolates demonstrated a more diverse population than the North/sub-Saharan isolates, where six out of seven 12S rRNA haplotypes were identified in the former region. *E. intermedius* (G6 genotype) was shown to be the most common species in the world camel population. In conclusion, camels showed to be an important intermediate host species in the MENA region with different patterns of genetic variation between the Middle East and Africa.

## Introduction

Tapeworms of the genus *Echinococcus*, causing a spectrum of infections known as echinococcosis, are members of the family Taeniidae. Cystic echinococcosis (CE) caused by the larval stages of *Echinococcus granulosus* sensu lato represents serious zoonotic infections in human and animals with a cosmopolitan distribution (1, 2). The life cycle involves dogs and other canids as definitive and domestic and wild ungulates as the intermediate hosts (3).

Extensive intraspecific variations have been documented within this species with significant epidemiological and clinical implications. *E. granulosus* genotype variation, may affect parasite life cycle and transmission patterns, host range and pathogenicity to humans (4, 5). Based on the biological and molecular genetic analyses using nuclear and mitochondrial DNA sequences 10 distinct genotypes with different host preferences have been identified for *E. granulosus* sensu lato and new nomenclature has been adopted for several genotypes (6) i.e., *E. granulosus* sensu stricto (G1–G3) with a wide range of intermediate hosts particularly sheep, goat and buffaloes, *E. equinus* (G4) of horses, *E. ortleppi* (G5) of cattle, *E. intermedius* (G6–G7) of camels and pigs, *E. canadensis* (G8 and G10) of Fennoscandian and subarctic cervids and *E. felidis* of African wild felid population (2).

According to Alvarez Rojas et al. about 88 and 11% of human CE infections are due to *E. granulosus* sensu stricto and *E. intermedius*, respectively (7). Therefore, camels are one of the most important intermediate hosts of the parasite especially in endemic regions of the Middle East and North Africa (MENA) where they play an important role in the transmission of *E. granulosus*. CE prevalence in camels has been estimated as 8–36% in different endemic countries (2, 8). In camels the presence of three *Echinococcus* species have been documented in different regions of the world, i.e., *E. granulosus* sensu stricto, *E. intermedius*, and *E. ortleppi* (2, 9). Several studies identified 100% of the parasites isolated from camels as *E. intermedius* (G6 genotype), however *E. granulosus* sensu stricto (G1–G3) has been shown to perpetuate in the camel-dog cycle as well (1, 10). Different studies showed 17.0–88.4% of camels harbored *E. granulosus* G1 genotype (11, 12). The nature and significance of *Echinococcus* variation in camels is poorly understood (6). Genotype G6 is more widespread in camels as a common intermediate host in the Middle East, Asia, and Africa, while G7 distribution has been reported among pigs and wild boars in Europe, the highly endemic Mediterranean areas and Central America (6). Both genotypes have been found to co-exist in Turkey, Argentina and Peru.

Mitochondrial DNA has been widely used for molecular epidemiological studies on helminth parasites mainly because of its conserved structure, mode of inheritance, and relatively high evolutionary rate. Currently one of the accepted markers for investigation of genetics and characterization of helminth parasites is mitochondrial 12S ribosomal RNA (12S rRNA) gene (13). The present study was conducted to provide a global outlook on the nature of genetic variation and haplotype distribution of *E. granulosus* sensu lato infecting camels using mitochondrial 12S rRNA gene sequence analysis.

## Materials and Methods

In this study all existing 12S rRNA gene sequences of camel isolates of *E. granulosus* sensu lato were retrieved form NCBI GenBank. To collect a dataset of 12S rDNA region, sequences data searched in and downloaded from NCBI nucleotide database (https://www.ncbi.nlm.nih.gov/nucleotide/) using keywords “Echinococcus,” “12S rRNA,” and “camel” with mitochondrial records filtration for sequences longer than 400 bp. The search strategy was performed as follows: ((((“Echinococcus”[Organism] OR Echinococcus[All Fields]) AND 12S[All Fields]) AND mitochondrion[filter]) AND (“Camelus dromedarius”[Organism] OR camel[All Fields])) AND (“400”[SLEN]: “14000”[SLEN]). All sequence features were manually checked to confirm the parasite host as camel.

In addition, liver and lung samples were collected from 57 camels slaughtered in the municipal abattoirs of Kerman, Qom, and Tehran provinces. The protoscoleces and/or germinal layer were aspirated from each cyst and were preserved in −20°C after three-time washing in sterile saline solution. Total genomic DNA (gDNA) was extracted from individual cyst samples using High Pure PCR template preparation kit (Roche Diagnostic, Germany) and was stored at −20°C for future molecular analysis.

A partial 450-bp fragment of mitochondrial 12S rRNA gene was amplified using specific primers as previously described by Rostami et al. 12SRF (5′-AGGGGATAGGACACAGTGCCAGC-3′) as the forward and 12SRR (5′-CGGTGTGTACATGAGCTAAAC-3′) as reverse primers (14). No template controls were included in each experiment. The PCR products were electrophoresed on 1% (w/v) agarose gel containing ethidium bromide. Moreover, a partial mitochondrial Cytochrome c oxidase subunit 1 (cox1) gene was amplified from individual isolates using the primer sets JB3/JB4.5 for an accurate distinction between genotypes within *E. granulosus* sensu stricto (15). All amplicons were sequenced by an ABI-3730XL capillary machine (Macrogen Inc., South Korea).

Reference sequences in this study were collected form reference nucleotide dataset (RefSeq) for representative *Echinococcus* species. Sequence data were trimmed, aligned, and edited manually in BioEdit software v.7.2 after equalizing each sample sequence length by the elimination of the PCR primers sequences and global sequence alignments using ClustalW algorithm (16). Phylogenetic analysis was performed with the best-fit model of nucleotide substitution on 12S rDNA sequences using the program Mega 7 software (17). Molecular phylogeny of *E. granulosus* isolates was determined by maximum likelihood method, and the corresponding phylogenetic tree was constructed. The heuristic tree search and tree topologies algorithm reliability test were estimated with bootstrap testing with 1,000 replicates.

Average evolutionary divergence of 12s rRNA sequences of all MENA camel *E. granulosus* sensu lato genotypes/haplotypes were also studied with MEGA7 software by using the Kimura 2-parameter model and the rate variation among sites was modeled with a gamma distribution (shape parameter = 1).

Population genetics analysis of haplotypes was performed on a consensus string of 420 positions in the aligned sequences for nucleotide diversity (π) and haplotype diversity (Hd) using DnaSP software (18). Associations between each haplotype of *Echinococcus* species form camel and their corresponding genotypes were inferred by constructing the TCS network using PopART software (19). The individual nucleotide sequence data reported in this study were submitted to the GenBank database.

## Results

Of 1,144 camel isolates of *E. granulosus* s.l. sequences retrieved from NCBI GenBank, 68 records for 12S rRNA gene were identified (Table 1). All 57 isolates were successfully PCR-sequenced on 12S rRNA gene and the sequences were submitted to the NCBI GenBank database under the accession numbers MH395757–MH395800 for 12sRNA and MH397251–MH397259 for cox1. Sequence analyses of 12S rRNA gene showed four genotypes including 35 G6 (61.4%), 15 G1 (26.3%), 6 G3 (10.5%), and one G5 (1.8%) representing three *Echinococcus* species, i.e., *E. intermedius, E. granulosus* sensu stricto, and *E. ortleppi*. Six camel isolates within G1-G3 complex were identified as G3 genotype using partial cox1 sequence analysis.

**Table 1:** The summary of global data on the frequency of *E. granulosus* sensu lato genotypes in camels (Camelus dromedarius) according to five major camel-rearing regions in the world.

**Region**	**Genotypes (No., %)**	**Country**	**No. each genotype/No. examined**	**Genotype (%)**	**Gene marker(s)**	**References**
North Africa		Algeria	6/6	G6 (100)	bg 1/3, cox1, nad1	(20)
			8/10 1/10 1/10	G6 (80) G1 (10) G2 (10)	cox1, nad1, act2 hbx2	(21)
	G1, G3 (55/289, 19)	Egypt	47/47	G6 (100)	12S rRNA	(22)
	G6, G7 (233/289, 80.6)		20/20	G6 (100)	nad1	(23)
			40/40	G6 (100)	12S rRNA	(24)
	G5 (1/289, 0.4)		26/28 1/28 1/28	G6 (92.9) G1 (3.57) G5 (3.57)	cox1, nad1, actin II	(5)
		Libya	5/5	G1 (100)	cox1	(25)
			83/83	G6 (100)	cox1, nad1	(26)
		Morocco	34/34	G1 (100)	cox1, nad1	(27)
		Tunisia	13/13	G1 (100)	cox1	(28)
			3/3	G6 (100)	ITS1, cox1	(29)
Sub-Saharan Africa		Ethiopia	9/12 3/12	G1–G3 (75) G6–G10 (25)	cox1	(30)
		Kenya	26/108 82/108	G1 (24.1) G6–G7 (75.9)	12S rRNA, cox1, nad1	(31)
	G1–G3 (55/530, 10.4)		17/100 83/100	G1–G3 (17.0) G6–G7 (83.0)	nad1	(11)
	G6–G7 (474/530, 89.4) Unidentified (1/530,0.2)		3/15 11/15 1/15	G1–G3 (20) G6–G7 (73.3) Unidentified (6.7)	nad1	(32)
		Mauritania	3/3	G6 (100)	bg 1/3, cox1, nad1	(33)
			1/1	G6–G7 (100)	12S rRNA	(34)
			17/17	G6 (100)	cox1, nad1, act2, hbx2	(21)
		Somalia	2/2	G6 (100)	cox1	(35)
		Sudan	35/35	G6–G7 (100)	12S rRNA, cox1, nad1	(31)
			207/207	G6–G7 (100)	12S rRNA, cox1, nad1	(36)
			30/30	G6 (100)	cox1, nad1	(37)
Central and East Asia	G1–G3 (1/2, 50) G6–G7 (1/2, 50)	China	1/1	G1 (100)	cox1	(35)
		Kazakhstan	1/1	G6 (100)	cob, nad3, nad1,cox1, cox2 and rrnS	(38)
Middle East		Iran	2/2	G6 (100)	cox1, nad1	(39)
			8/32 24/32	G1 (25.0) G6 (75.0)	ITS1	(40)
	G1–G3 (144/318, 45.3)		5/19 8/19 6/19	G1 (26.3) G3 (42.1) G6 (31.6)	cox1, nad1	(41)
	G6–G7 (172/318, 54.1)		9/26 17/26	G1 (34.6) G6 (65.4)	ITS1	(42)
	G5 (2/318, 0.6)		4/9 2/9 3/9	G1 (44.4) G3 (22.2) G6 (33.3)	nad1, cox1	(43)
			38/43 5/43	G1 (88.4) G6 (11.6)	cox1, nad1, atp6, 12S rRNA	(44)
			4/14 4/14 5/14 1/14	G1 (28.6) G3 (28.6) G6 (35.7) G5 (7.1)	cox1, nad1	(9)
			25/100 4/100 71/100	G1 (25) G3 (4) G6 (71)	cox1	(13)
			15/57 6/57 35/57 1/57	G1 (26.3) G3 (10.5) G6 (61.4) G5 (1.8)	12S rRNA, cox1	Present study
		Oman	11/15 4/ 15	G1 (73.3) G6 (26.7)	12S rRNA	(45)
		Turkey	1/1	G1 (100)	cox1, ITS1	(46)
South Asia	G1–G3 (5/5, 100)	Pakistan	5/5	G1 (100)	cox1	(47)
Total				G1-G3 (260/1144, 22.7) G6–G7 (880/1144, 76.9) G5 (3/1144, 0.3) Unidentified (1/1144, 0.1)		

**Figure 1:**
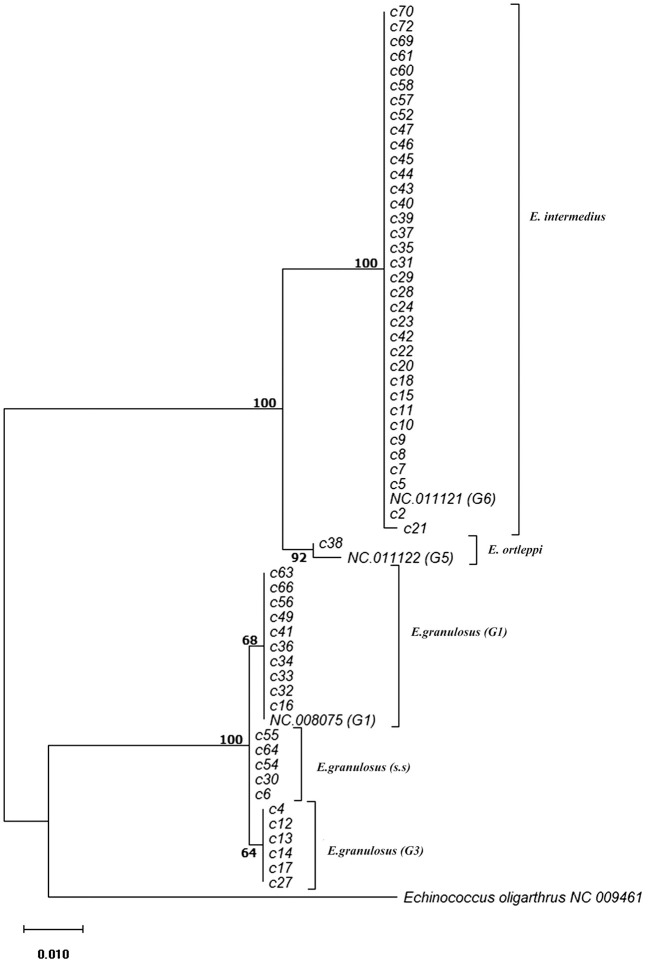
Phylogenetic relationships of camel isolates of *Echinococcus granulosus* sensu lato based on 12S mitochondrial rRNA sequences based on Hasegawa-Kishino-Yano model. The heuristic search was obtained automatically by applying Neighbor-Join and BioNJ algorithms to a matrix of pairwise distances estimated using the Maximum Composite Likelihood (MCL) approach, and then selecting the topology with superior log likelihood value. The percentage of trees in which the associated samples clustered together is shown next to the branches. The tree is drawn to scale with branch lengths based on the number of substitutions per site. The analysis involved 60 nucleotide sequences. All positions containing gaps and missing data were eliminated. There was a total of 420 positions in the final dataset. Evolutionary analyses were conducted in MEGA7.

Phylogenetic analysis of 12S rRNA sequence data from 57 camel isolates compared with reference genotypes of *E. granulosus* s.l. is shown in Figure 1. Four genotypes, G1, G3, G6, and G5 exhibit distinct clusters along with corresponding reference sequences representing all of the sequences determined in the present study. The haplotype diversity for 57 camel isolates from Iran (categorized into six haplotypes) was calculated as Hd = 0.6050 (±0.063). However, the haplotype diversity for 68 camel records from MENA region (categorized into seven haplotypes) was estimated at Hd = 0.5540 (±0.064).

**Figure 2:**
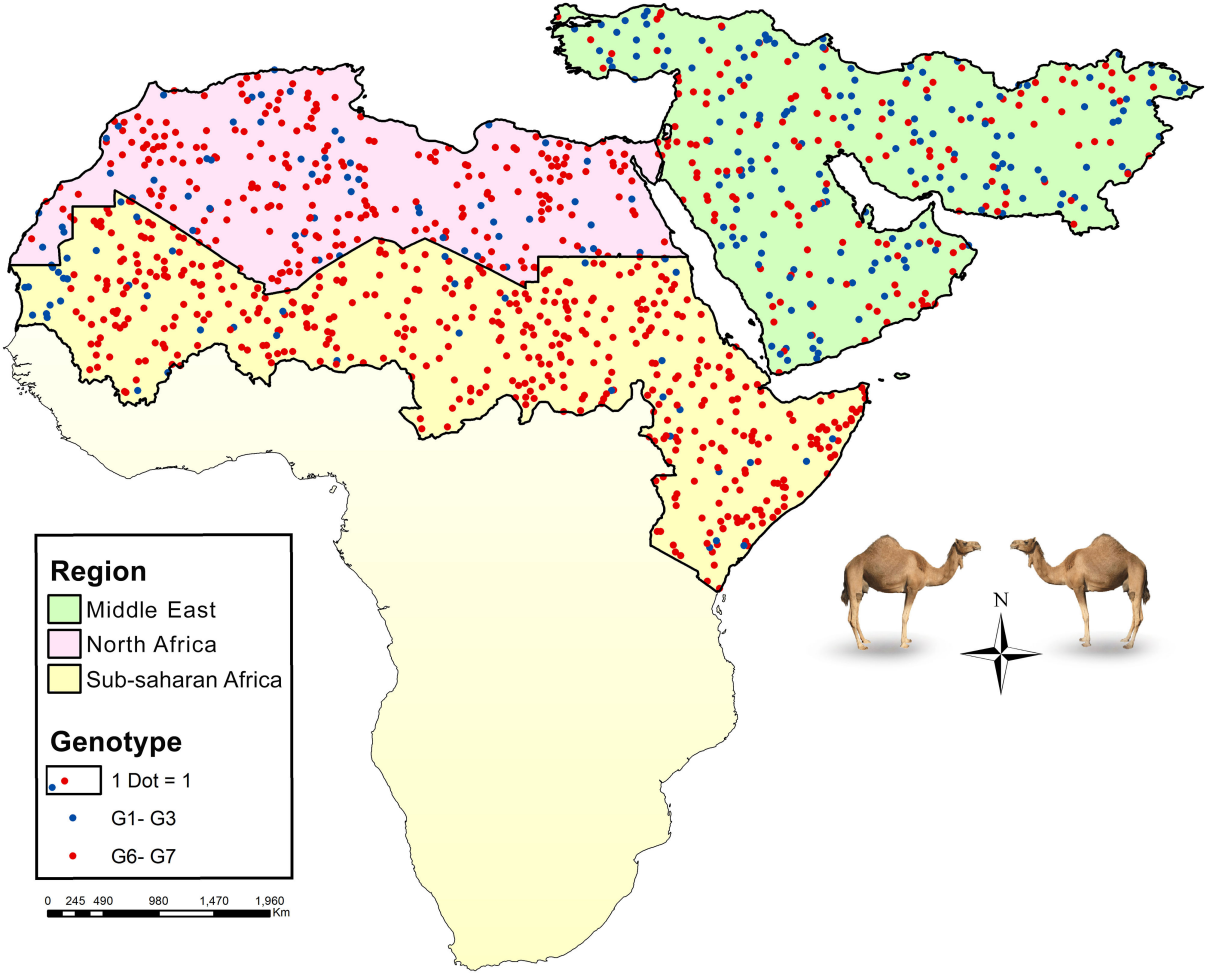
Map showing the relative frequency distribution of two major *Echinococcus* species infecting camels, *E. granulosus* sensu stricto (G1–G3) and *E. intermedius* (G6), in three main regions of camel CE transmission, the Middle East (green), North Africa (purple), and part of sub-Saharan Africa (yellow). Each dot represents a camel isolate.

Global data on the frequency of *E. granulosus* s.l. genotypes in camels according to five major camel-rearing regions in the world, i.e., the Middle East, North Africa, sub-Saharan Africa, East/Central Asia, and South Asia are summarized in Table 1. As it is shown, three different genotype patterns could be obtained in camels infected by *E. granulosus* sensu lato. *E. intermedius* (G6, camel strain) retained its dominance in camels from sub-Saharan Africa, so that about 90% of *Echinococcus* species isolated from camels were *E. intermedius*. In the Middle East, *E. granulosus* sensu stricto replaced *E. intermedius*, interestingly half of *Echinococcus* species isolated from camels in this region have been identified as *E. granulosus* sensu stricto. The situation in North Africa is something in between, ~80% of the parasites are G6 camel strain (Figure 2, Table 1).

**Figure 3:**
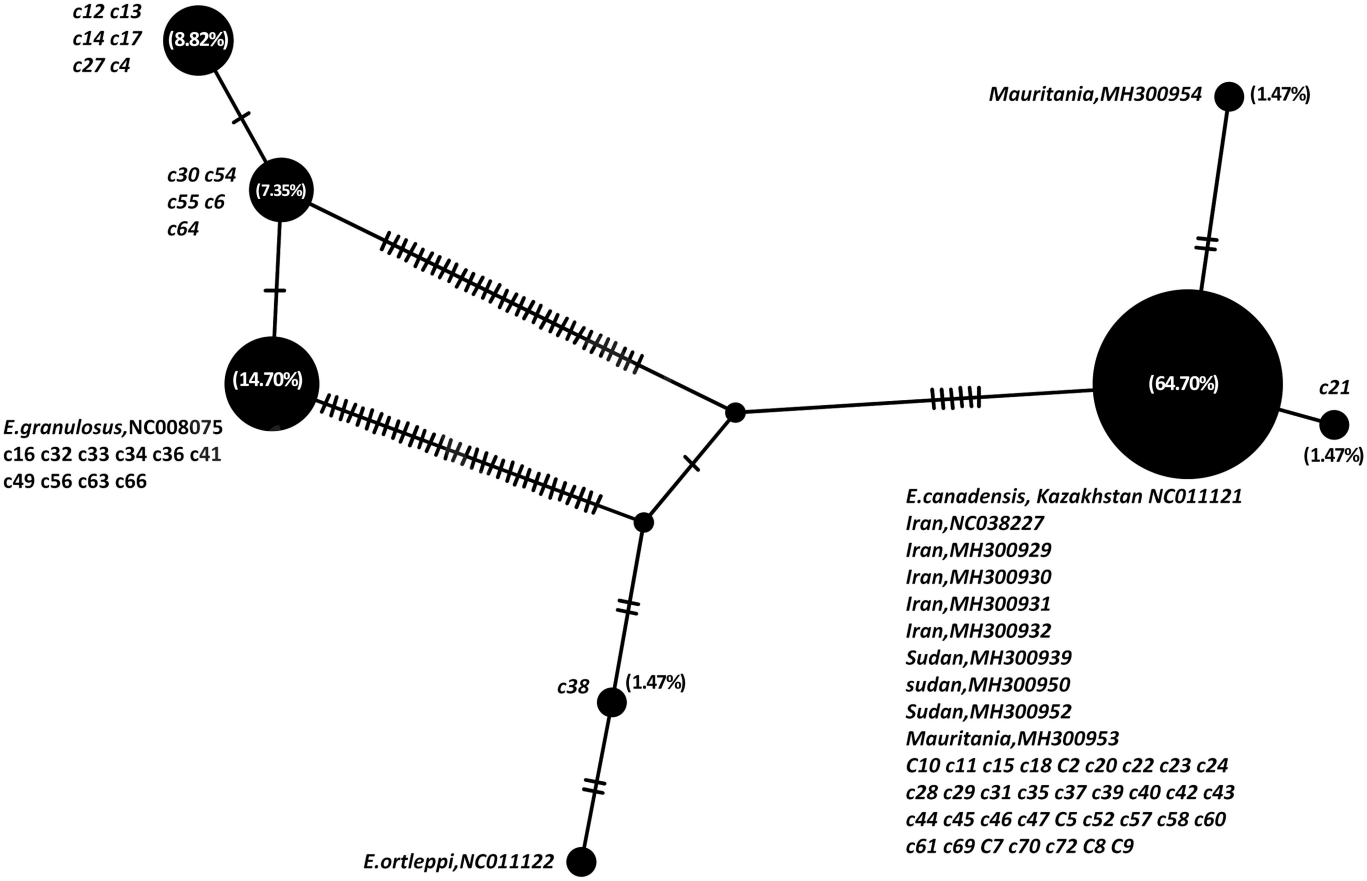
Haplotype network analysis of *Echinococcus granulosus* sensu lato of partial 420 bp of 12S rRNA gene on all available camel records from the Middle East and Africa, based on statistical parsimony. The size of the circles indicates the frequency of the haplotypes. The nucleotide accession numbers and each individual isolate code in the present study are indicated next to each circle. Numbers in parenthesis are the percent frequency of the isolates in each haplotype.

The parsimony-based haplotype network analysis on all MENA records, indicated that *E. granulosus* sensu lato isolates were clustered into four major groups (Figure 3). The G6 genotype (NC011121) corresponds to the most commonly represented *E. granulosus* haplotype in camels originated from Central Asia, the Middle East and Africa, followed by G1 (NC008075) representing most of the isolates from Iran.

**Figure 4:**
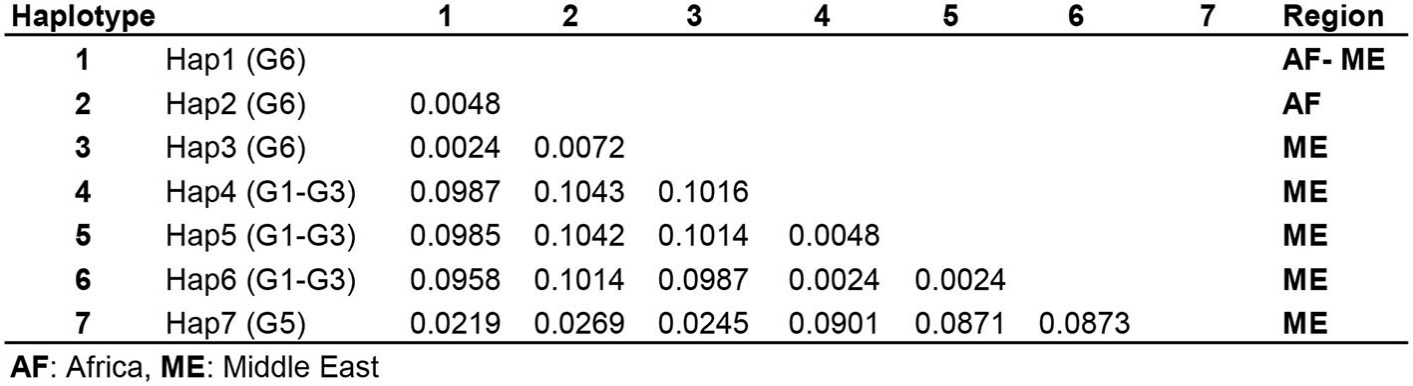
Pairwise comparison of nucleotide sequence differences (%) in mitochondrial 12S rRNA gene among seven available camel haplotypes within *Echinococcus granulosus* sensu lato.

The number of base substitutions per site over all 420 positions of 12S rRNA partial sequence pairs were shown as 0.2–0.7% and 0.4% within the Middle Eastern and sub-Saharan *E. intermedius* (G6) isolates, respectively. The average overall distance of all MENA *E. granulosus* s.l. isolates was 0.6% (Figure 4).

## Discussion

Control of cystic echinococcosis is complicated due to the fact that CE transmission occurs through different definitive/intermediate hosts systems (48). In this regard cystic echinococcosis of dromedaries has been received less attention among different livestock species. Camels play an essential role in the epidemiology and transmission of CE in the Middle East and North Africa. Little is known about the nature and significance of genetic variation of camel isolates of *E. granulosus* s.l. in the endemic areas. Most of the studies investigated a limited number of camel isolates which are not representatives of *E. granulosus* population in camels. In this study we present a global outlook of the significance of different species/genotypes of camel isolates of *E. granulosus* sensu lato in the molecular epidemiology of CE in the main camel breeding regions of the world using all available genetic markers including both nuclear and mitochondrial regions.

It has been already shown that 12S rRNA gene is a suitable marker for differentiating genotypes of E. granulosus, nonetheless few 12S rRNA nucleotide data are available in GenBank, particularly from camel CE. In the present study we investigated 12S rRNA gene diversity of 57 camel isolates of *E. granulosus* s.l. from Iran. The findings indicate that G6 genotype is the most common genotype (61.4%) in this Iranian dromedary population. The results also revealed the existence of three other genotypes including G1 (26.3%), G3 (10.5%), and G5 (1.8%).

The study showed that *E. granulosus* s.l. perpetuates in a camel-dog cycle mostly comprising of G6 genotype (*E. intermedius*) and several other less prevalent strains. Both species, *E. granulosus* s.s. and *E. intermedius* are believed to be overlapped in their cycle so that sheep and camels have contributed to the transmission of both species/genotypes. However, there are controversies on the contribution of the genotypes in each individual intermediate host species. Several studies showed the dominant species infecting camels is *E. granulosus* s.s. G1 genotype (10, 25, 28, 30, 43–45, 49), while several other studies reported higher frequency of *E. intermedius* in camels than *E. granulosus* s.s. (4, 9, 11, 20, 22, 25, 26, 29, 31, 33, 34, 36, 50, 51). In addition, sylvatic transmission of CE among camel populations should also be considered. Wild carnivores particularly wolves and golden jackals have long been considered as epidemiologically important definitive hosts in MENA region (52, 53). Regarding the nature of camel husbandry, the animals are usually roaming freely in vast geographical areas outside human dwellings during their long life span, therefore it is quite probable for camels to get involved in the sylvatic life cycle of *E. granulosus* sensu lato.

Assuming that G6 parasites had primarily perpetuated in wild camels across the old world, this is probably an indication of different forces of infection with G1 sheep strain in different camel-rearing areas from the Iranian plateau to the Maghreb and sub-Saharan Africa. With the Middle East has already been occupied by *E. granulosus* s.s. G1 genotype (sheep strain) as the dominant genotype in the region, exerting remarkable forces of infection on the camels regularly infected by G6 genotype.

The camel domestication took place some 8000 years later than that of sheep and goat (54).

It is believed that camels were first domesticated in the Fertile Crescent and Arabian Peninsula in 2000–1000 BCE. Increasing trade of basic commodities including incense, myrrh and frankincense mainly transported by the camels across the region, facilitated the dispersion of *E. granulosus* s.s. We could not find any significant different nucleotide diversity among isolates from Middle East and sub-Saharan Africa. This may be due the fact that the emergence of domestic camel-dog cycle of *E. granulosus* s.l. following camel domestication is a relatively recent event, therefore the development of significant haplotype diversity within the camel isolates is not expected in a short period of time. Similar molecular epidemiological picture has been demonstrated in sheep-dog cycle in which the universal sheep strain (G1 genotype) is the dominant variant, however other genotypes including G3 and G6 are perpetuating in the G1 endemic areas.

It should be noted that the sample size in several camel studies is small and the findings are not conclusive (Table 1). Mitochondrial gene sequences are highly informative markers for molecular taxonomy and phylogenetic investigation of helminth parasites. Cytochrome c oxidase subunit 1 and 12S ribosomal RNA genes have been extensively used for genetic classification of tapeworms. However, few studies on *E. granulosus* 12S rRNA sequences have been performed on African camel isolates and obviously more 12S rRNA data is required from this important endemic region. It has been shown that 12S rRNA gene presents higher sequence variability than cox1, therefore it is assumed that the camel isolates of *E. granulosus* s.l. in Africa present even more homogeneous population than those of the Middle East (13).

In addition, the majority of the studies have used small partial fragments of mitochondrial genes. Using longer sequences in phylogeographical studies provides more reliable information on the molecular epidemiology of CE in camels (6). This study presents the second evidence of G5 presence in the Middle East camels. This suggests that *E. ortleppi*, the common cattle strain, could also be transmissible through a camel-dog cycle in the region. Previous studies in MENA indicated that camels usually harbor highly fertile/highly viable hydatid cysts reaching >90% fertility and >80% viability (14, 28, 55, 56). However, interestingly in our study few protoscoleces were found in the camel hydatid cyst harboring G5 genotype (*E. ortleppi*) and DNA was extracted from the cyst germinal layer. This is an indication of low susceptibility of camels to *E. ortleppi* and can explain the low prevalence of G5 in camels, however, to obtain a more comprehensive picture of the global epidemiology of *E. ortleppi*, further studies are required on the molecular epidemiology of camel CE in the MENA region.

## Conclusion

Global data on the frequency of *E. granulosus* s.l. genotypes in camel populations of major camel-rearing regions of the world revealed a changing pattern of genotype distribution between the Middle East and African isolates. *E. intermedius* (G6 genotype) was identified as the most common species in camels, however the contribution of this species in camel populations in various areas of MENA is significantly different across the region from the Iranian plateau to sub-Saharan Africa. Mitochondrial 12S rDNA study of camel isolates of *E. granulosus* s.l. revealed significant species/genotype diversity. Camel isolates of *E. granulosus* in the MENA region present moderate genetic diversity with the Middle East isolates demonstrating a more diverse population than the North/sub-Saharan isolates, where three species, four genotypes and six different 12S rRNA haplotypes were identified in the region.

Camels are an important intermediate host species in Iran, harboring different species and genotypes of *E. granulosus* s.l. throughout their long lifetime. More in-depth large-scale studies using multiple large fragments of mitochondrial/nuclear gene sequences are required to elucidate the significance and actual contribution of camels in CE epidemiology.

## Data Availability Statement

The datasets generated for this study can be found in online repositories. The names of the repository/repositories and accession number(s) can be found in the article/supplementary material.

## Ethics Statement

This project was reviewed and approved by Research Review Committee of the National Institutes for Medical Research Development (NIMAD), No. 971237. The animals were slaughtered as part of the normal daily practice in the abattoirs.

## Author Contributions

MF, MM, and SR: conceptualization and study design. MD, SH, SN, and SR: data curation. MD, MM, SN, and SR: data analysis and laboratory experiments. MF: funding acquisition. MM, SH, SR, and MF: data validation. MD, MM, SH, SN, SR, and MF: writing—original draft preparation. All authors contributed to the article and approved the submitted version.

## Conflict of Interest

The authors declare that the research was conducted in the absence of any commercial or financial relationships that could be construed as a potential conflict of interest. The reviewer AS declared a past co-authorship with one of the author MF to the handling editor.
